# Volatile Compound Profiling by HS-SPME/GC-MS-FID of a Core Olive Cultivar Collection as a Tool for Aroma Improvement of Virgin Olive Oil

**DOI:** 10.3390/molecules22010141

**Published:** 2017-01-14

**Authors:** Lourdes García-Vico, Angjelina Belaj, Araceli Sánchez-Ortiz, José M. Martínez-Rivas, Ana G. Pérez, Carlos Sanz

**Affiliations:** 1 Department of Biochemistry and Molecular Biology of Plant Products, Instituto de la Grasa, CSIC, Campus University Pablo de Olavide, Ctra. Utrera km 1, Building 46, 41013-Seville, Spain; lourdesgarcia@ig.csic.es (L.G.-V.); araceli.sanchez.ortiz@juntadeandalucia.es (A.S.-O.); mrivas@cica.es (J.M.M.-R.); agracia@cica.es (A.G.P.); 2 IFAPA, Centro Alameda del Obispo, Menendez Pidal s/n, 14004-Cordoba, Spain; angjelina.belaj@juntadeandalucia.es

**Keywords:** *Olea europaea* L., virgin olive oil, core collection, volatile compounds, quality

## Abstract

Virgin olive oil (VOO) is the only food product requiring official sensory analysis to be classified in commercial categories, in which the evaluation of the aroma plays a very important role. The selection of parents, with the aim of obtaining new cultivars with improved oil aroma, is of paramount importance in olive breeding programs. We have assessed the volatile fraction by headspace-solid-phase microextraction/gas chromatography-mass spectrometry-flame ionization detection (HS-SPME/GC-MS-FID) and the deduced aroma properties of VOO from a core set of olive cultivars (Core-36) which possesses most of the genetic diversity found in the World Olive Germplasm Collection (IFAPA Alameda del Obispo) located in Cordoba, Spain. The VOO volatile fractions of Core-36 cultivars display a high level of variability. It is mostly made of compounds produced from polyunsaturated fatty acids through the lipoxygenase pathway, which confirms to be a general characteristic of the olive species (*Olea europaea* L.). The main group of volatile compounds in the oils was six straight-chain carbon compounds derived from linolenic acid, some of them being the main contributors to the aroma of the olive oils according to their odor activity values (OAV). The high level of variability found for the volatile fraction of the oils from Core-36 and, therefore, for the aroma odor notes, suggest that this core set may be a very useful tool for the choice of optimal parents in olive breeding programs in order to raise new cultivars with improved VOO aroma.

## 1. Introduction

The positive impact on human health [1,2] and its sensory properties are among the main aspects that make virgin olive oil (VOO) attractive to consumers. With respect to the sensory properties, the volatile fraction of VOO determines its aroma, which is mainly characterized by a mixture of green and fruity odor notes that make it a unique edible oil. The aroma of VOO is synthesized during the oil extraction process when enzymes and substrates come together as a result of the olive fruit crushing. Six straight-chain carbons (C6), aldehydes, C6 alcohols, and their corresponding esters are the most important compounds in VOO aroma [3,4,5]. The synthesis of these compounds depends mainly on the level of lipoxygenase (LOX) activity and the availability of substrates to be catabolized through the LOX pathway during the process of obtaining the oil [6,7]. This synthesis starts from polyunsaturated fatty acids, such as linoleic (LA) and linolenic (LnA) acids, by the consecutive action of LOX, hydroperoxide lyase (HPL), and alcohol dehydrogenase (ADH) enzymatic activities to produce, respectively, the 13-hydroperoxide derivatives of the polyunsaturated fatty acids, the C6 aldehydes, and the C6 alcohols [3,8,9,10]. Finally, the C6 alcohols can be substrates for ester production catalyzed by alcohol acyltransferase (AAT) enzymatic activities [3,11]. Five straight-chain carbon compounds (C5) are also important to VOO aroma [5] and seem to be synthesized through another branch of the LOX pathway involving the production of a 1,3-pentene allylic radical that can dimerize to pentene dimers (PD), or to form C5 alcohols by reaction with a hydroxyl radical. These C5 alcohols may be converted into C5 carbonyl compounds via enzymatic oxidation by ADH.

VOO is the only food product that requires a sensory analysis to be classified in commercial categories. This classification is ruled by the European Official Regulations for olive oil [12] and is carried out by certified test panels, in which the evaluation of taste (pungency and bitterness) and aroma play a very important role. In this regard, Aparicio and Morales [13] developed the statistical sensory wheel (SSW) for VOO to understand the connection between volatile compounds and odor characteristics in the oil. The SSW clusters into different sectors the sensory attributes of VOO together with the volatile compounds labeled with a particular sensory note, those in the green or ripe fruit sectors being the most important to VOO aroma. This sensory perception of a volatile compound depends on its odor activity value (OAV), the ratio between the concentration of the volatile compound and its odor threshold, so that only volatile compounds with an OAV higher than one contribute to VOO aroma. However, it should be mentioned that OAV values are only indicative of the contribution of the volatiles to the VOO overall aroma, since they do not take into account enhancement or suppression effects of different odorants and other non-volatile components present in the oils.

The wide genetic patrimony is one of the key features of the olive tree. Much emphasis has been laid on the collection and conservation of olive genetic resources leading to the establishment of ex-situ germplasm banks. In this sense, the World Olive Germplasm Collection (WOGC), located in Cordoba, Spain, is an international reference due to the high number of accessions included and their high degree of identification and evaluation. Molecular markers and agronomical traits were used to study the patterns of genetic diversity and underlying genetic structure among olive accessions in the WOGC [14] and, as a result, a core set of 36 olive cultivars (Core-36), representing 10% of the whole germplasm bank, was established, which holds most of this genetic diversity (Table 1). Considering the significance of the aroma to VOO quality, the purpose of the present work was to assess the volatile fraction and deduced aroma properties of the oils from the Core-36 cultivars, most of them never previously studied, to provide a useful tool for the selection of optimal parents in olive breeding programs aiming at improving VOO flavor quality.

## 2. Results and Discussion

**Figure 1:**
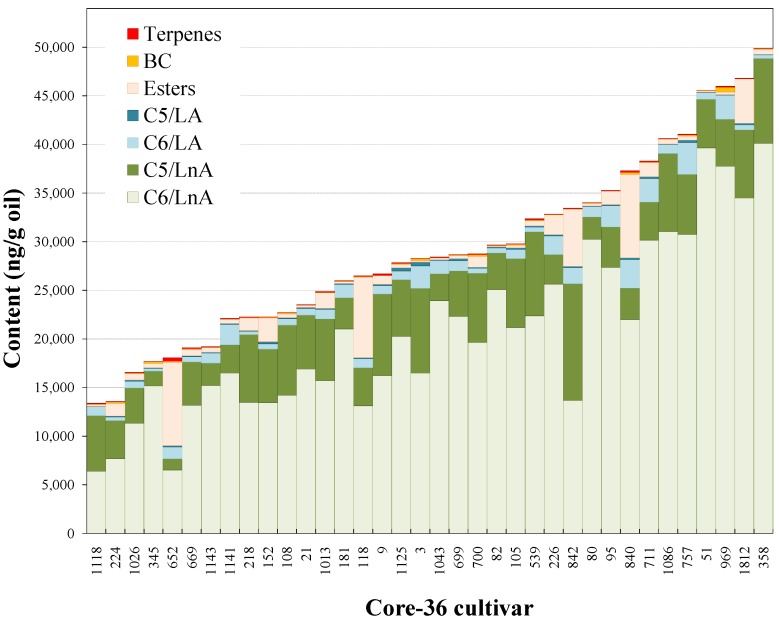
Content of the main groups of volatile compounds in the oils of the Core-36. Fruits at the turning stage were hand-picked during years 2008–2012.

Oils from the Core-36 cultivar collection exhibited a high level of variability regarding the volatile compound contents (Figure 1 and Table 2). Most of the VOO volatile compounds are synthetized through the LOX pathway [3], and they may be classified into different groups regarding chain length (C6 and C5), the polyunsaturated fatty acid origin (LnA or LA), and the origin of the esters (LOX esters). Additionally, a group of compounds, derived from amino acids, carrying a branched-chain (BC) structure and a terpene, seem also to contribute significantly to the volatile fraction of the oils. Only hand-picked, sound fruits and mild operation conditions were used in this study; as a consequence the oils were devoid of compounds responsible for the off-flavors, such as carboxylic acids or aliphatic C8-C11 carbonyls and alcohols [15].

The main group of volatile compounds in the oils of the Core-36 cultivars are derived from linolenic acid (LnA), the six straight-chain carbon compounds (C6/LnA), and account, on average, for 4–400 times higher content than those of the rest of volatile compounds in the oils (Table 2). The range of variability for the content of C6/LnA compounds was 6382–40,104 ng/g oil. This range was greater than that found by Luna et al. [29] for oils from 39 different cultivars. However, it was slightly lower than those observed for the segregating progeny of the cross of two of the cultivars from the collection, Picual and Arbequina (500–51,990 ng/g oil) [30]. The C6/LnA aldehyde group of compounds was the most abundant. (*E*)-hex-2-enal was the most important component (averaging 89% of C6/LnA), showing a mean content of 18,518 ng/g oil and a range of 4916–37,643 ng/g oil (Table 2) in good agreement with the average value found for the volatile fractions of the progeny of the Picual × Arbequina cross [30]. This compound might be the main contributor to the aroma of the olive oils produced from the cultivars included in Core-36 due to the high content in the oils, which seems to be a general characteristic of VOO [30], and the relatively low odor threshold (Table 2).

The C5/LnA group of compounds exhibited also contents as high as those of the C6/LnA. Among them, pentene dimers showed to be 79% of the C5/LnA content on average. However, they seem to have a minor role in the VOO aroma according to their estimated odor thresholds. Thresholds were estimated (13,500 ng/g oil) from the structurally-related C6-C10 dienes [23]. However, the rest of C5/LnA compounds seem to be important contributors to VOO aroma because of their OAVs. Pent-1-en-3-one and the pent-2-en-1-ols are particularly notable in good agreement with data found by Aparicio et al. [31]. The content of pent-1-en-3-one in the oils obtained from all of the cultivars of the Core-36 was higher than its odor threshold (Table 2), but its green pungent odor note in the VOO aroma is considered unpleasant [5]. On the contrary, pent-2-en-1-ols provide green fruity odor notes to the oil, which are associated to oils extracted from fresh olives harvested at the optimal ripening stage. Most of the cultivars from Core-36 collection have (*Z*)-pent-2-en-1-ol contents below their threshold concentration, which suggests that, in general, this component is of little significance for the VOO aroma. On the contrary, all of the cultivars included in the Core-36 collection produce oils whose (*E*)-pent-2-en-1-ol contents are higher than their odor threshold (Table 2).

The content of esters synthesized through the LOX pathway (LOX esters) present in the oils of the Core-36 collection ranged 29–8414 ng/g oil, and showed an average value of 1447 ng/g oil. Esters present in the oils are very important for the oil commercial grading and from a consumer perspective, as they are responsible for the fruity odor notes in this product. Among them, (*Z*)-hex-3-en-1-yl acetate seems to contribute to VOO aroma (OAV > 1) in nearly 60% of the Core-36 collection (Table 2). However, the contents of hexyl acetate and (*E*)-hex-2-en-1-yl acetate were relevant to VOO aroma in only a few cultivars included in Core-36. Data for the LOX esters largely agree with those observed for the progeny of the cross of Picual × Arbequina [30].

Low concentrations of branched-chain volatile compounds (BC) were found in the oils (Table 2), showing a mean value of 112 ng/g oil and a variability range of 39–521 ng/g oil. However, they could play a relevant role in the oil aroma, except for 2-methyl-butan-1-ol, which was found below its odor threshold in the oils from the Core-36 collection. This BC compound is generally related to the fusty defects of VOO aroma [32]. On the contrary, BC aldehydes 2 and 3-methyl-butanal, situated in the SSW ripe fruit sector as reported by Aparicio and Morales [13], seem to be important contributors to VOO aroma according to their contents (Table 2). All of the oils from the Core-36 cultivar collection displayed OAV values for these two BC aldehydes higher than one.

Terpenes also presented a significant, though low, content in the oils of the Core-36 cultivar collection, limonene being its main representative. The limonene contents ranged in the interval 0–336 ng/g oil and had a mean value of 54 ng/g oil. In general, it seems not to be an important contributor to VOO aroma since just one of the cultivars had an OAV above one for this terpene (Table 2).

Pearson’s correlation coefficients were calculated in order to study the relationship between the main groups of volatile compounds found in the oils from the Core-36 cultivar collection (Table S1 in Supplementary Materials). As expected, the total content of volatile compounds in the oils correlated significantly with the major group of volatile compounds in the oils (C6). Particularly remarkable is the strong and very significant correlation with the C6/LnA aldehydes (*r* = 0.91). On the contrary, the rest of the C6 groups showed a significant, but moderate, correlation (*r* = 0.35, 0.46, and 0.32). By contrast, most of the C5 compounds displayed a low correlation coefficient except for the pentene dimers that carried a moderate level of correlation (*r* = 0.38) with total volatiles. Esters and BC compounds displayed no correlation with the total oil volatile compound contents. These data are in good agreement to those found for the oils of the segregating population of the Picual × Arbequina cross [30], except for the fact that the relationship of the C5 compounds with total volatiles was moderate to high in the latter. A conceivable explanation might be that the parents of the cross, Picual and Arbequina, are cultivars whose oils contain average to high contents of C5 compounds compared to the rest of the Core-36 cultivars.

Despite the significant impact of LOX esters and BC aldehydes on VOO aroma, it is remarkable their low correlation coefficients in relation to the total contents of volatiles. Actually, no high correlations were found with any other groups of compounds. BC compounds do not share any synthetic pathways with the rest of the volatile compounds, so these findings are logical from a metabolic point of view, however they are not for the LOX esters. Esters were moderately correlated with the C6/LA alcohol (*r* = 0.42) but to a much lower level with their main precursors, the C6/LnA alcohols (*r* = 0.23), suggesting either a limitation of AAT activity or that the synthesis of alcohol by ADH during VOO production is generally reduced in the Core-36 cultivars, as demonstrated to occur to two cultivars from this collection, Arbequina and Picual [33]. In this sense, although a high and significant correlation between C6/LnA and C6/LA alcohols (*r* = 0.73) was found, suggesting the same biochemical origin, however, a weak correlation was observed between the content of C6/LnA aldehydes and their metabolic products, the C6/LnA alcohols (*r* = 0.15). This low correlation was also noticed for the C6/LA aldehyde contents and those of the C6/LA alcohol (*r* = 0.39).

On the other hand, a significant but weak correlation was observed between the contents of C5/LnA carbonyls and C5/LnA alcohols (*r* = 0.36), quite similar to that found for each of these groups of compounds with the contents of pentene dimers (*r* = 0.50 and 0.29, respectively). According to these results, these groups of compounds might be metabolically related, as described also for the oils from the progeny of the Picual × Arbequina cross [30] and, consequently, supporting the hypothesis that the synthesis of C5 alcohols and pentene dimers occurs at the same time catalyzed by a LOX activity [34]. Moreover, it seems that the pentene dimers are only produced from LnA, since no significant correlations were found for pentene dimers and the carbonyl and alcohol derived from LA (C5/LA).

The cultivars included in the Core-36 collection could not be grouped in terms of harvest year or geographical origin when analyzed by a principal component analysis (PCA) using all of the volatile compounds as the variables (Figure S1 in Supplementary Materials) despite the fact that Belaj et al. [14] did find some grouping between cultivars from the different geographical origins after PCA of the whole olive bank. Thus, most of the variability among the Core-36 volatile profiles seems to correspond exclusively to the cultivar.

**Figure 2:**
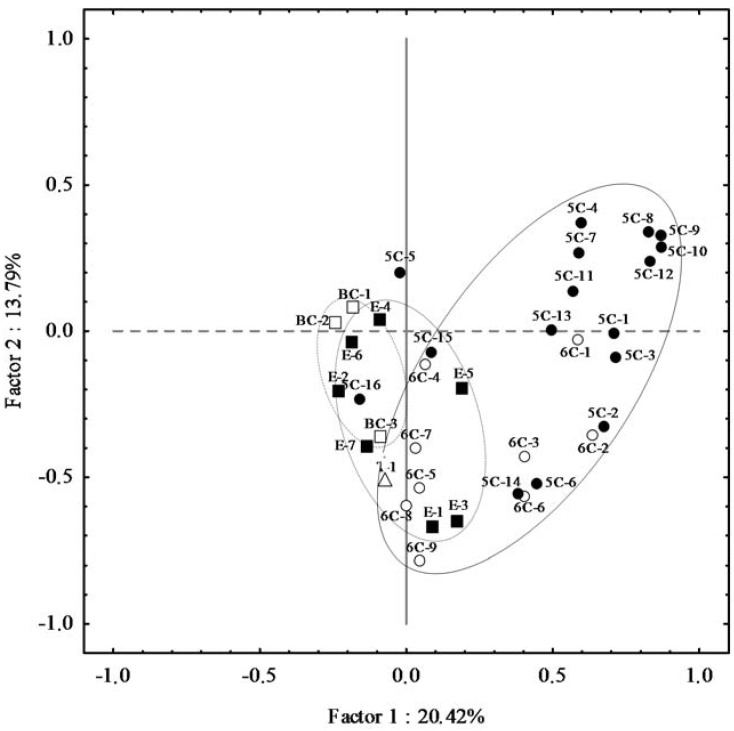
Vector distribution of the main volatile compounds in the oils of the Core-36.

In order to explain relationships between the different groups of volatile compounds in the oils of the Core-36 cultivar collection, a PCA was performed (Figure 2). The first two PCs explain 34.21% of the total variance, 20.42% for PC1 and 13.79% for PC2, reasonably similar to what found for the content of the volatile compounds in the oils of the progeny of the Picual × Arbequina cross (25.73% and 11.47% for PC1 and 2, respectively) [30]. The C6 aldehydes and C5 compounds derived from LnA clustered together between the first and fourth quadrants, and their variances are mostly explained by PC2 (Figure 2). Isomers (*Z*)-pent-2-en-1-ol (5C-5) and (*E*)-pent-2-en-1-ol (5C-6) are markedly separated in the plot, indicating that probably they have different origins. According to the grouping, (*E*)-pent-2-en-1-ol would have a biochemical origin, synthesized through the LOX pathway (homolytic branch), however the origin of (*Z*)-pent-2-en-1-ol is unknown. The C6 compounds (6C-8, 6C-9) and the C5 alcohol (pentan-1-ol, 5C-16) derived from LA clustered also separated from the LnA-derived C6 aldehydes and C5 compounds. This was also observed when examining the oil volatile profile from the progeny of the Picual × Arbequina cross [30]. The synthesis of these compounds could be ascribed to a mixture of chemical and biochemical oxidation processes, the latter through the LOX pathway. Actually, Kalua et al. [26] proposed that hexanal formation during VOO extraction maybe due to both enzymatic and non-enzymatic synthetic methods.

C6 alcohols derived from both LA and LnA clustered along the PC2 negative axis clearly distanced from the rest of compounds synthesized by the LOX pathway (Figure 2). The distance in the plot from their precursors in the LOX pathway could be explained by the weak correlation displayed by these groups of compounds mentioned above, probable consequence of ADH inactivation during the oil extraction process [33]. The esters clustered along the PC2 negative axis, overlapping the space of their metabolic precursors synthesized through the LOX pathway, which suggests that, on average, cultivars from the Core-36 collection seem not to have limited AAT activity and that only the limitation of ADH activity during the oil extraction process would be responsible for the moderate correlation between esters and the C6 alcohols (Table S1 in Supplementary Materials). The BC compounds clustered away from the rest of compounds were synthetized by the LOX pathway, indicative of a different metabolic origin.

**Figure 3:**
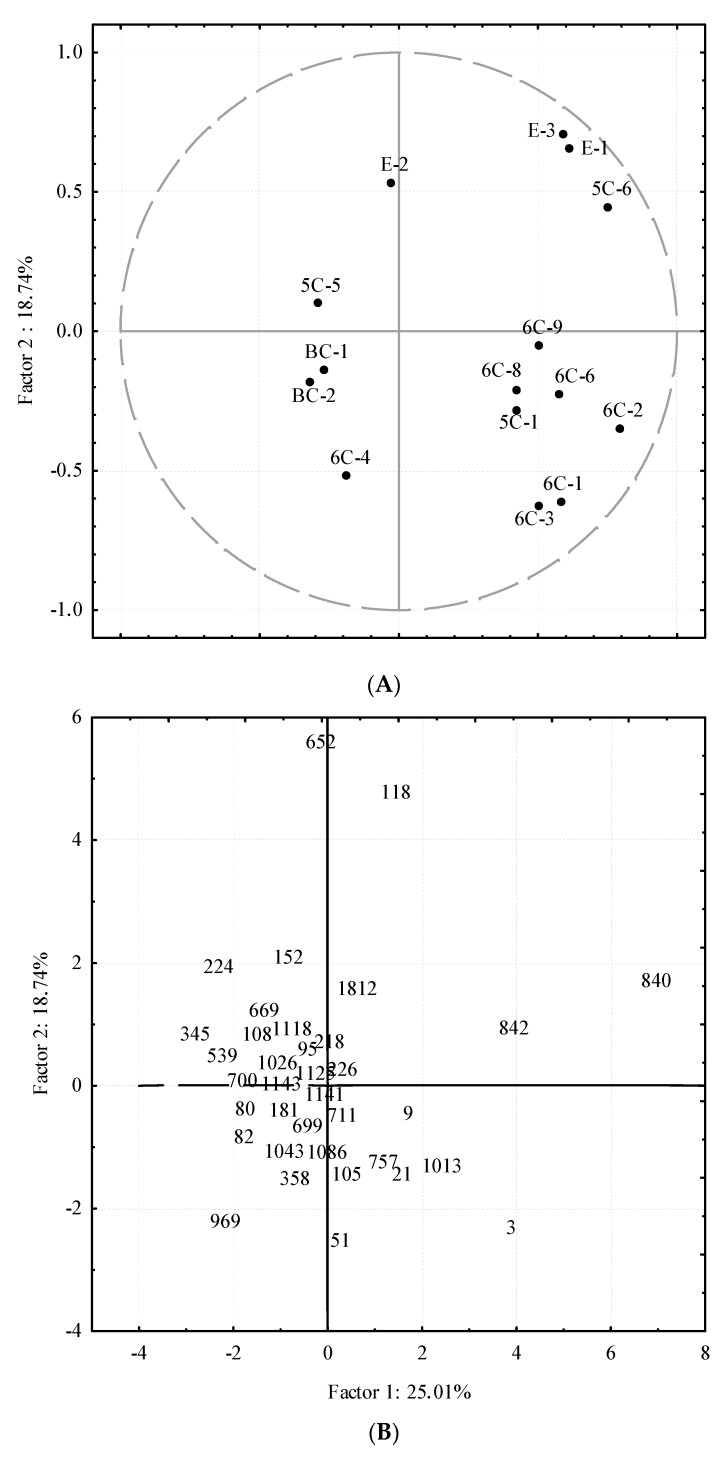
Bi-plot of selected volatile compounds contributing to the aroma (OAV > 1) of the Core-36 oils. (**A**) Vector distribution of the volatile compounds. Codes for the volatile compounds are listed in Table 2; and (**B**) distribution of the cultivars from the collection. References for the cultivars are listed in Table 1.

Among the main volatile compounds in the oils from the Core-36 collection, only some of them have OAVs greater than one, thus, theoretically contributing to the oil aroma (Table 2). In this regard, PCA was carried out taking as variables only those volatile compounds having OAV > 1 in at least 5% of the cultivars (Figure 3). Apart from hexan-1-ol (6C-9) and pent-1-en-3-one (5C-1), responsible for unpleasant odor notes [5,14,32], most compounds contribute with pleasant odor notes to the oils. As shown in Figure 3, the first two PCs explain 44% of the total variance. The vector distribution of volatile compounds may distinguish pleasant and unpleasant areas in the Core-36 distribution plot. Thus, those cultivars producing oils with a high content of the desired fruity esters are located in the first quadrant of the plot. Meanwhile, cultivars featuring high contents of (*E*)-hex-2-enal (6C-4) are positioned in the third quadrant as well as those having high contents of the branched-chain aldehydes (BC-1 and BC-2), delivering green-fruity odor notes in the oils according to Aparicio et al. [31]. On the contrary, cultivars whose oils have high contents of the unpleasant hexan-1-ol (6C-9) and pent-1-en-3-one (5C-1) are located along the bisector of the fourth quadrant.

In conclusion, the Core-36 cultivar collection shows a high level of variability in terms of the volatile fraction of the oils and, presumably, of the aroma quality. This aroma variability and the high genetic diversity suggest that the Core-36 cultivar collection may be useful for the choice of optimal parents for olive breeding programs with the aim of finding new cultivars with improved oil aroma. Furthermore, due to the availability of both genotypic and phenotypic data, Core-36 may be used as the training population for olive genomic selection to estimate model parameters that will subsequently be employed to calculate genomic-estimated breeding values of candidate breeding lines from their genotypic data [35].

## 3. Materials and Methods

### 3.1. Plant Material

Thirty-six olive (*Olea europaea* L.) cultivars corresponding to the Core-36 olive set (Table 1) established and maintained at WOGC (IFAPA Alameda del Obispo in Cordoba (Andalucia, Southern Spain) were studied. This collection is the result of a joint project between different institutions (CAP-UCO-IFAPA) and includes cultivars collected in Spain and international prospecting surveys and/or provided by different scientific institutions. Trees, two per accession, were cultivated in the same conditions at the experimental orchards of IFAPA (Alameda del Obispo, Cordoba, Spain) using standard culture practices, including irrigation supply by in-line drippers to avoid water stress of plants (290 mm, Mars-October), containing N (80 kg/year/ha), P_2_O_5_ (40 kg/year/ha), and K_2_O (110 kg/year/ha). The harvest was carried out by hand during five consecutive years (2008–2012) at the turning stage (2.5) to compare the volatile fraction of the oils from the different cultivars.

### 3.2. Olive Oil Extraction

Oil was extracted from batches of 2–3 kg of olive fruits using a laboratory oil extraction plant (Abencor, Comercial Abengoa, S.A., Seville, Spain) that mimics the industrial VOO production according to Martinez et al. [36]. Fruit milling was carried out with a stainless steel hammer mill working at 3000 rpm and a 5 mm sieve. The Abencor thermo-beater was used for the malaxation step for 30 min at 28 °C. Finally, paste centrifugation was performed in a basket centrifuge for 1 min at 3500 rpm. Oils were decanted, filtered through paper, and stored under a nitrogen atmosphere at −20 °C until analysis.

### 3.3. Analysis of Oil Volatile Compounds

Extraction and analysis of oil volatile compounds were performed by means of HS-SPME/GC-MS-FID according to Perez et al. [30]. Samples for volatile analyses (0.5 g) were prepared in 10-mL vials and placed in a vial heater at 40 °C for a 10 min equilibration time. Headspace volatile compounds were adsorbed onto a headspace-solid-phase microextraction (SPME) fiber divinylbenzene/carboxen/polydimethylsiloxane (DVB/Carboxen/PDMS) 50/30 µm (Supelco Co., Bellefonte, PA, USA). Sampling time was carried out at 40 °C for 50 min. Desorption of volatile compounds was completed into the GC injector. Compound identification was carried out by means of a 7820A/GC-5975/MSD system (Agilent Technologies, Santa Clara, CA, USA), equipped with a DB-Wax capillary column (60 m × 0.25 mm i.d., film thickness, 0.25 µm: J and W Scientific, Folsom, CA, USA). Operating conditions were as follows: He as carrier gas at 1 mL/min flow rate; injector in splitless mode at 250 °C; column held for 6 min at 40 °C and then programmed at 2 °C/min to 168 °C; the mass detector ran in the electronic impact mode at 70 eV, the source temperature was set at 230 °C and the mass spectra were scanned at 2.86 scans/s in the *m*/*z* 40–550 amu range. Compound identification was performed by matching against the Wiley/NBS Library, and by GC retention time against standards. For quantitative purposes, the volatile fraction was analyzed three times on a HP-6890 GC equipped with a FID detector (Agilent Technologies, Santa Clara, CA, USA) using also a DB-Wax capillary column operated under the following conditions to reproduce the same retention times for volatile compounds than those of the 7820A/GC-5975/MSD system: N_2_ as carrier gas at 17 psi constant pressure; injector and detector at 250 °C; column held for 6 min at 40 °C and then ramped up at 2 °C/min to 168 °C. For quantification, calibration curves for each compound were made in re-deodorized high-oleic sunflower oil. Limits of quantitation (≥three times LOD) and concentration ranges were established according to typical contents in virgin olive oil volatile fractions. Linear regression curves were found for all the compounds with regression coefficients higher than 0.989. A blank containing no oil and a mixture of volatile standards, used at the beginning and regularly during the sample analyses, were run as controls. The volatile compounds were clustered into different groups according to the number of carbons (C6 and C5 compounds) and esters originated through the LOX pathway and the branched-chain (BC) volatile compounds coming from the amino acid metabolism. 

### 3.4. Statistical Analysis

The program STATISTICA (Statsoft Inc., Tulsa, OK, USA) was used for data treatment. Pearson’s correlation coefficients were calculated for the volatile compounds or groups of volatile compounds, and principal component analysis (PCA) was used to study the associations among the volatile compounds from the Core-36 cultivar collection.

## Figures and Tables

**Table 1:** Olive cultivars included in the Core-36 collection.

Cultivar	WOGC Reference	Country	Mediterranean Geographical Area
Abbadi Abou Gabra	842	Syria	East
Abou Kanani	840	Syria	East
Abou Satl Mohazam	1043	Syria	East
Arbequina	1086	Spain	West
Barnea	711	Israel	East
Barri	1026	Syria	East
Chemlal de Kabilye	118	Algeria	Centre
Dokkar	539	Turkey	East
Forastera de Tortosa	652	Spain	West
Frantoio	80	Italy	Centre
Grappolo	181	Italy	Centre
Jabali	1118	Syria	East
Kalamon	105	Greece	Centre
Klon-14-1812	1812	Albania	Centre
Koroneiki	218	Greece	Centre
Leccino	82	Italy	Centre
Llumeta	226	Spain	West
Maarri	1125	Syria	East
Majhol-1013	1013	Syria	East
Majhol-152	152	Syria	East
Manzanilla de Huercal Overa	757	Spain	West
Manzanilla de Sevilla	21	Spain	West
Mari	1143	Iran	East
Mastoidis	345	Greece	Centre
Mavreya	699	Greece	Centre
Megaritiki	108	Greece	Centre
Menya	669	Spain	West
Morrut	224	Spain	West
Myrtolia	700	Greece	Centre
Picual	9	Spain	West
Picudo	3	Spain	West
Pinonera	969	Spain	West
Shengeh	1141	Iran	East
Temprano	358	Spain	West
Uslu	95	Turkey	East
Verdial de Velez-Malaga-1	51	Spain	West

**Table 2:** Variability and distribution of volatile contents (ng/g oil) in the oils from the WOGC Core-36 accessions. Oils were obtained from fruits at the turning stage, which were hand-picked during years 2008–2012.

Group	Subgroup	Volatile Compound	Codes	Min	Max	Mean	Median	Cultivars OAV > 1	Odor Threshold (ng/g Oil)	References
C6/LnA	C6/LnA aldehydes	(*E*)-hex-3-enal	6C-1	78	581	236	194	3	450	[16]
		(*Z*)-hex-3-enal	6C-2	52	2180	589	395	36	1.7	[17]
		(*Z*)-hex-2-enal	6C-3	175	1059	587	622	28	* 424	[17,18]
		(*E*)-hex-2-enal	6C-4	4916	37,643	18,518	16,443	36	424	[17]
	C6/LnA alcohols	(*E*)-hex-3-enol	6C-5	4	105	29	20	0	1500	[19]
		(*Z*)-hex-3-enol	6C-6	72	4306	571	402	3	1100	[17]
		(*E*)-hex-2-enol	6C-7	39	559	211	175	0	5000	[20]
C6/LA	C6/LA aldehyde	hexanal	6C-8	195	2175	809	684	36	300	[17]
	C6/LA alcohol	hexan-1-ol	6C-9	39	1740	361	183	7	400	[19]
C5/LnA	C5/LnA carbonyls	pent-1-en-3-one	5C-1	68	1020	425	358	36	0.73	[17]
		(*Z*)-pent-2-enal	5C-2	4	78	24	20	0	* 300	[18,20]
		(*E*)-pent-2-enal	5C-3	22	184	73	61	0	300	[20]
	C5/LnA alcohols	pent-1-en-3-ol	5C-4	29	339	157	155	0	400	[21]
		(*Z*)-pent-2-en-1-ol	5C-5	14	730	111	45	4	250	[22]
		(E)-pent-2-en-1-ol	5C-6	98	1085	336	281	36	* 250	[18,22]
	Pentene dimers	pentene dimer-1	5C-7	32	583	266	243	0	* 13,500	[23]
		pentene dimer-2	5C-8	33	461	213	197	0	* 13,500	[23]
		pentene dimer-3	5C-9	135	1978	950	890	0	* 13,500	[23]
		pentene dimer-4	5C-10	127	2892	1066	1017	0	* 13,500	[23]
		pentene dimer-5	5C-11	34	1599	375	325	0	* 13,500	[23]
		pentene dimer-6	5C-12	37	1676	723	698	0	* 13,500	[23]
		pentene dimer-7	5C-13	68	1789	569	524	0	* 13,500	[23]
C5/LA	C5/LA carbonyls	pentan-3-one	5C-14	18	335	79	71	0	7000	[24]
		pentanal	5C-15	9	197	52	40	0	240	[20]
	C5/LA alcohol	pentan-1-ol	5C-16	3	46	13	11	0	470	[25]
Esters	LOX esters	hexyl acetate	E-1	8	4390	513	113	5	1040	[25]
		(*E*)-hex-2-en-1-yl acetate	E-2	14	1876	131	39	3	* 200	[17,18]
		(*Z*)-hex-3-en-1-yl acetate	E-3	0	5285	876	253	22	200	[17]
	non-LOX esters	methyl acetate	E-4	5	30	15	15	0	200	[26]
		ethyl acetate	E-5	4	94	20	13	0	940	[20]
		methyl hexanoate	E-6	6	282	35	22	0	* 520	[27]
		ethyl hexanoate	E-7	8	78	27	21	0	* 520	[27]
BC	Aldehydes	3-methyl-butanal	BC-1	9	263	41	30	36	5.4	[17]
		2-methyl-butanal	BC-2	7	182	29	17	36	5.2	[17]
	Alcohol	2-methyl-butan-1-ol	BC-3	7	146	33	23	0	480	[20]
Terpenes		limonene	T-1	0	336	54	40	1	250	[28]

* Odor thresholds estimated from isomers [18] or structurally close compounds [23,27].

## Supplementary Materials

The following are available online at www.mdpi.com/1420-3049/22/1/141/s1, Table S1 and Figure S1.
