# Supplementary material for: Volatile Compound Profiling by HS-SPME/GC-MS-FID of a Core Olive Cultivar Collection as a Tool for Aroma Improvement of Virgin Olive Oil
Source: Molecules. 2017 Jan 14;22(1):141. doi: 10.3390/molecules22010141 (PMC6155863; doi:10.3390/molecules22010141)
Supplement: Supplementary file 1 [file molecules-22-00141-s001.pdf]

Supplementary Materials: Volatile Compound Profiling by HS-SPME/GC-MS-FID of a Core Olive Cultivar Collection as a Tool for Aroma Improvement of Virgin Olive Oil

Lourdes Garcia-Vico, Angjelina Belaj, Araceli Sanchez-Ortiz, Jose M. Martinez-Rivas, Ana G. Perez and Carlos Sanz

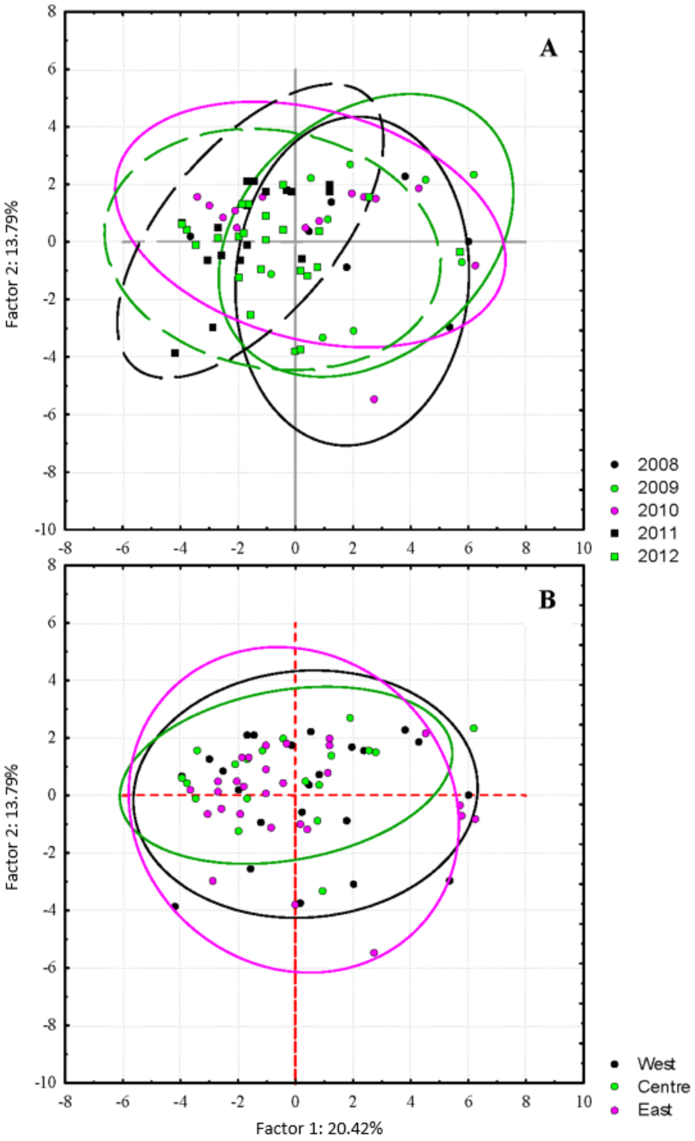

Figure S1. Distribution of the cultivars from the Core-36 olive collection from the World Olive Germplasm Collection at Cordoba, Spain according to the crop year (**A**) or area of diffusion (**B**). Prediction ellipses are displayed for each year or area. Principal component analysis was carried out using all of the volatile compounds as variables.

Table S1. Pearson's correlation coefficients among the main classes of volatile compounds found in the oils from the WOGC Core-36 accessions.

	**C6/LnA Aldehydes**	**C6/LnA Alcohols**	**C6/LA Aldehyde**	**C6/LA Alcohol**	**C5/LnA Carbonyls**	**C5/LnA Alcohols**	**Pentene Dimers**	**C5/LA Carbonyls**	**C5/LA Alcohol**	**LOX Esters**	**Non-LOX Esters**	**BC Aldehydes**	**BC Alcohol**	**Terp enes**	**Total C6 Volatiles**	**Total C5 Volatiles**
C6/LnA alcohols	0.15															
C6/LA aldehyde	*** 0.41	** 0.34														
C6/LA alcohol	0.13	*** 0.73	*** 0.39													
C5/LnA carbonyls	−0.06	* 0.24	0.02	0.04												
C5/LnA alcohols	* −0.28	0.10	−0.17	0.09	** 0.36											
Pentene dimers	0.18	0.21	−0.10	−0.10	*** 0.50	* 0.29										
C5/LA carbonyls	−0.11	*** 0.51	−0.01	*** 0.42	** 0.35	* 0.24	0.12									
C5/LA alcohol	0.18	0.18	* 0.29	0.17	−0.11	−0.17	−0.10	0.08								
LOX esters	−0.15	0.23	0.12	*** 0.42	0.03	*** 0.55	−0.07	* 0.24	−0.09							
non-LOX esters	0.03	0.00	0.09	0.17	−0.20	-0.08	−0.13	0.07	0.19	0.03						
BC aldehydes	−0.11	−0.09	0.05	−0.02	−0.12	0.00	−0.11	−0.04	0.00	−0.07	−0.03					
BC alcohol	−0.15	0.21	0.15	*** 0.44	−0.15	0.08	−0.13	* 0.26	−0.07	* 0.25	0.21	*** 0.39				
Terpenes	−0.16	0.14	0.23	* 0.27	0.04	* 0.28	−0.17	0.13	0.00	*** 0.41	0.18	0.09	* 0.24			
Total C6 volatiles	*** 0.94	* 0.31	*** 0.51	** 0.35	−0.04	-0.11	0.16	0.01	0.18	0.17	0.04	−0.13	−0.03	−0.01		
Total C5 volatiles	0.11	** 0.24	−0.11	−0.06	*** 0.61	*** 0.45	*** 0.98	0.21	−0.12	0.04	−0.15	−0.11	−0.11	−0.10	0.12	
Total volatiles	*** 0.91	** 0.35	*** 0.46	** 0.32	0.11	0.00	** 0.38	0.06	0.14	0.17	0.02	−0.14	−0.05	−0.03	*** 0.97	** 0.35

Marked correlations are significant at: * $p < 0.05$. ** $p < 0.01$. *** $p < 0.001$.
